# Evaluation of the use of microbubbles in the ultrasound assessment of the axilla in breast cancer patients

**DOI:** 10.1186/bcr3765

**Published:** 2015-11-05

**Authors:** Nisha Sharma, Isobel Haigh, Rebecca Millican-Slater, Benjamin Dessauvagie

**Affiliations:** 1 St James's Hospital, Leeds, UK

## Introduction

Contrast-enhanced ultrasound of the axilla can be used to identify the axillary sentinel lymph node. We introduced this into our practice in 2013. During the study period there was an upgrade of our US equipment. The purpose of our audit was to see the negative predictive value of CEUS biopsy of the SLN.

## Methods

This was a retrospective audit. In total, 110 patients with invasive breast cancer were identified at the breast MDT. The US core biopsy, surgical sentinel node biopsy and subsequent axillary histology were documented.

## Results

CEUS was successful in identifying the first draining lymph node in 88.1% (97/111). Eighty-three of 97 cases (86%) had a definitive biopsy (B2−B5) result with 13 being malignant and 69 were benign. Fifteen were non-diagnostic with B1 core biopsy. The prevalence of axillary metastases at surgery was 31% (30/97) (22 macrometastases, six micrometastases and two isolated tumour cells) of which 42% were detected by CEUS, with 100% specificity. Two of the 30 cases were in palpable, non-sentinel nodes. The negative predictive value of CEUS with core biopsy is 80% but 90% if only macrometastases are included.

## Conclusion

CEUS and biopsy is a promising technique for reducing the false negative rate of imaging at the time of SLNB. Our numbers are small and we had a transition to different equipment during the study, but it is felt that reproducible data comparable with Cox et al. [1] is achievable.
